# Family Environment and Childhood Obesity: A New Framework with Structural Equation Modeling

**DOI:** 10.3390/ijerph14020181

**Published:** 2017-02-13

**Authors:** Hui Huang, Che Wan Jasimah bt Wan Mohamed Radzi, Hashem Salarzadeh Jenatabadi

**Affiliations:** Department of Science and Technology Studies, Faculty of Science, University of Malaya, Kuala Lumpur 50603, Malaysia; huanghui@siswa.um.edu.my (H.H.); jenatabadi@um.edu.my (H.S.J.)

**Keywords:** family food security level, childhood obesity, public health, child’s environment, household environment, structural equation modeling

## Abstract

The main purpose of the current article is to introduce a framework of the complexity of childhood obesity based on the family environment. A conceptual model that quantifies the relationships and interactions among parental socioeconomic status, family food security level, child’s food intake and certain aspects of parental feeding behaviour is presented using the structural equation modeling (SEM) concept. Structural models are analysed in terms of the direct and indirect connections among latent and measurement variables that lead to the child weight indicator. To illustrate the accuracy, fit, reliability and validity of the introduced framework, real data collected from 630 families from Urumqi (Xinjiang, China) were considered. The framework includes two categories of data comprising the normal body mass index (BMI) range and obesity data. The comparison analysis between two models provides some evidence that in obesity modeling, obesity data must be extracted from the dataset and analysis must be done separately from the normal BMI range. This study may be helpful for researchers interested in childhood obesity modeling based on family environment.

## 1. Introduction

As the largest developing country around the world in the past two decades, China has witnessed a rapid increase in childhood obesity rates along with the fast economic growth [1]. Martinson et al. [2] addressed the notable rise in childhood overweight and obesity that has mainly been dependent on China’s public health. Zhang and Wang [3] stated that the prevalence of overweight and obesity increased from 1.79% and 1.66% in 1985 to 31.12% and 20.11% in 2014 for boys and girls, respectively. In another study in China, researchers found that the obesity rates among children 7–12 years old living in a city, township and rural area were 10.3%, 8.5% and 5.5% respectively [4]. Moreover, based on a 2016 World Health Organization report, around 170 million children worldwide below the age of 18 were suffering from the physical and psychological consequences of overweight or obesity [5]. According to recent studies, Vinturache et al. [6] and Baek et al. [7], for instance, believe that obesity has emerged as one of the most substantial public health concerns in the last two decades. It is now identified as a severe threat to society due to its rapidly expanding prevalence. Therefore, focus should be geared toward preventive efforts against childhood obesity, as problems with a child’s body status are mostly caused by unusual weight gain.

Family food security level [8,9], parental feeding behaviour [10], the child’s food intake [11,12] and parental socioeconomic status [13,14,15] are deemed the most essential and recognizable indicators in childhood obesity modeling literature. The influence of socioeconomic status as an independent determinant or mediator of behaviour further adds intricacy to our understanding of obesity and related behaviours [16,17]. Some studies suggest that family food security level and its output as well as the child’s health are antecedents of childhood obesity [8]. Beside cultural and genetic factors influencing a child’s food structure, the family food security level together with eating behaviour has a pivotal role. During the childhood years, parents model their own feeding behaviour, which affects the child’s eating habits [18]. Several studies concerning the impacts of parental feeding behaviour [19,20] have illustrated there is a relationship between parental feeding behaviour, the child’s food intake and child obesity. Despite the outcomes, many variations regarding the impact of different behaviours still exist. Parental restriction of their children’s amount of food intake is the feeding behaviour most dependably related to a higher risk of becoming overweight.

Moreover, regarding the impact of the family environment on childhood obesity, some reviews have concentrated on some child behaviours and obesity [21] with the child’s sleeping behaviour being one. The relationship between sleep quality and obesity has been stressed in some studies [22,23]. Chen et al. [24] reviewed seventeen studies on sleep and child obesity, and found that children with a shorter average amount of sleep had a 58% higher risk of obesity issues than peers with adequate amounts of sleep. Parallel to what has been found regarding lack of sleep in children, excessive technology use (mobile phones, tablets, TV, etc.) was linked to child obesity [10,25]. This similarity can be explained by the high concurrence of excessive technology use and sleep problems. As a basic aspect of the family environment, the parental weight status [26] and physical activity [27] appeared to be vital predictors of the increase in childhood obesity.

Unfortunately, there are very few studies investigating the impact of children behaviours like technology use, average sleep time and physical activity considering the family environment and especially the family food security level with parental socioeconomic status, feeding behaviour and weight in controlling children’s obesity. Nevertheless, research on the simultaneous integration of the interrelationships among three well-known concepts, i.e., parental socioeconomic status, parental feeding behaviour and the child’s food intake into one model remains scarce.

Moreover, in the majority of previous modeling studies, different regression equations have been used to measure and estimate the suggested indicators affecting childhood obesity indicators [28,29,30]. However, obesity regression modeling does not facilitate estimating causal and indirect effects in a single and integrated equation. Multicollinearity exists among indicators too, which is a major factor that reduces model efficiency and power and cannot be ignored. In other words, it is possible to illustrate that an entire model has good fit, but it is not possible to confirm whether there are valid outputs from any individual research predictor in a multiple regression model that includes correlated predictors. Besides, estimating the overall impact of both observed and unobserved indicators that lead to a child’s weight is risky.

**Figure 1:**
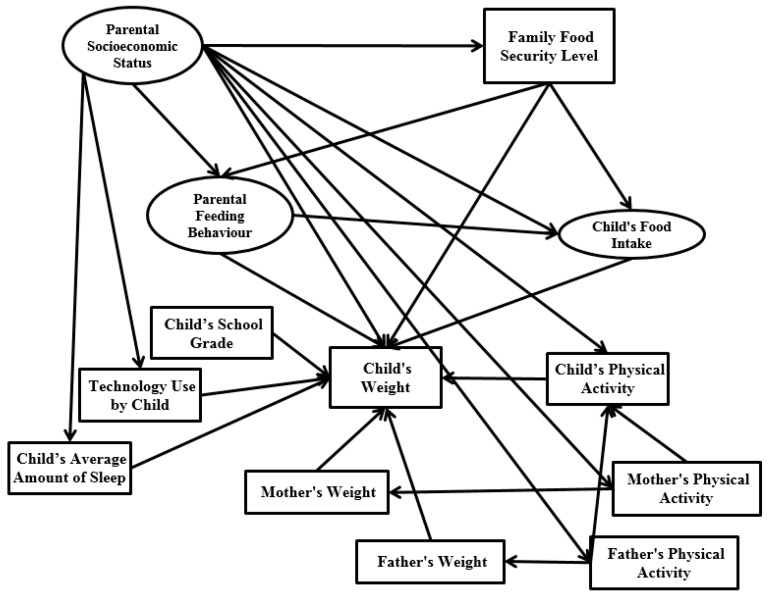
Research framework.

Therefore, the aim of this study is to introduce an integrated model capable of providing an overall evaluation of latent and observed variables by applying a multi-combination of the three basic concepts (parental socioeconomic status, parental feeding behaviour and child’s food intake) with the concept of SEM. Therefore, the aim of this study is to introduce an integrated model capable of providing an overall evaluation of latent and observed variables by applying a combination of the three basic concepts (parental socioeconomic status, parental feeding behaviour and child’s food intake) within the SEM framework. To better understand the impact of putative factors on childhood obesity, we examine the relationship among child technology use, the child’s average amount of sleep, the child’s school grade, the child’s physical activity and the parents’ physical activity, and the child’s weight. Figure 1 illustrates the conceptual framework of the research model. In the research framework, parental socioeconomic status (initial independent variable) and the child’s school grade are exogenous variables, the child’s weight (main dependent variable) acts as an endogenous variable, and the remaining variables are both endogenous and exogenous.

## 2. Materials and Methods

### 2.1. Measurements

#### 2.1.1. Parental Socioeconomic Status

In this study, parental socioeconomic status was measured as the initial independent variable including seven indicators. Six of the indicators are the mother’s education, father’s education, mother’s income, father’s income, mother’s work experience and father’s work experience. The question “How long have the parents been married?” was added and is representative of the parents’ marital length.

The parents’ ages were classified into four groups: 30 years old or younger, 31 to 40 years old, 41 to 50 years old, and over 50 years old. With respect to education level, the responses obtained were categorized as “Less than high school”, “High school”, “Diploma”, “Bachelor” and “Master or Ph.D.”. The respondents were asked about parental income status, and the answers were denoted by “Less than RMB2000 per month”, “RMB2001–RMB3000 per month”, “RMB3001–RMB4000 per month”, “RMB4001–RMB5000 per month” and “more than RMB5000 per month”. The respondents were asked about parental work experience and the responses were denoted by “less than 5 years”, “5–10 years”, “11–15 years”, “16–20 years” and “more than 20 years”. The last question in the socioeconomic part is related to the length of the parents’ marriage and responses were indicated by “less than 2 years”, “2–4 years”, ‘5–7 years”, “8–10 years” and “more than 10 years”.

#### 2.1.2. Family Food Security Level

Family food security level was measured based on Bickel and Nord [31] study, which included 18 standard questions. Appendix A presents the standard questions for measuring family food security level.

#### 2.1.3. Parental Feeding Behaviour

The six indicators include restricting, monitoring, rewarding, pressuring, controlling and modeling based on Birch and Fisher [32] child feeding questionnaire (CFQ). All scales assess the frequency of every parental feeding behaviour on a 5-point Likert scale (“never” to “always”) or a greater agreement (“disagree” to “agree”), with higher scores indicating more frequent use of a specific feeding behaviour.

#### 2.1.4. Child’s Food Intake

We measured the child’s food intake based on Kröller and Warschburger [33] study. The seven indicators include consumption of fruits, vegetables, whole grain products, sweets, chips, soft drinks and fast food. Parents indicated on a six-point scale (“never”, “seldom”, “sometimes”, “most of the time”, “always” and “several times a day”) how often their children eat certain foods.

#### 2.1.5. BMI

BMI is a measure of relative size based on the mass and height of an individual [34]:

(1) $$\mathrm{BMI}\;\left(\mathrm{Metric}\;\mathrm{Method}\right)=\frac{\left(\mathrm{weight}\;\mathrm{in}\;\mathrm{kilograms}\right)}{{\mathrm{height}\;\mathrm{in}\;\mathrm{meters}}^{2}}$$

BMI applies differently to children. It is calculated in the same way as for adults but is then compared to typical values for other children of the same age. Instead of comparing against fixed underweight and overweight thresholds, the BMI is compared against the percentile of children of the same gender and age [35].

A BMI below the 5th percentile represents underweight and above the 95th percentile indicates obese. Children with a BMI between the 85th and 95th percentile are considered overweight [35]. Table 1 shows the BMI categories for children.

Every primary school student has a “health card” with some information like weight and height. We asked the parents to provide their children’s weight and height based on the cards and we calculated each child’s BMI based on the above formula.

#### 2.1.6. Control Variables

Regarding the frequency of parental physical activity, the respondents were asked “how many times a week on average do you do physical activity?” The responses to this question consist of four categories denoted by “none”, “1 or 2 times a week”, “3 or 4 times a week” and “more than 4 times a week”. The child’s average number of sleeping hours per day were also grouped in “less than 7 h per day”, “7 to 8 h per day”, “8 to 9 h per day” and “more than 9 h per day”. As for the average number of h per day the child uses technology, the respondents were asked “how many h per day on average does your child use technology?” The responses to this question consist of four categories: “less than one hour per day”, “1 to 2 h per day”, “3 to 4 h per day” and “more than 4 h per day”. The child’s school grade can be from one to six. The child’s physical activity per week consists of “none”, “1 or 2 times per week”, “3 or 4 times per week” and “more than 4 times per week”.

### 2.2. Why Using SEM?

SEM is strongly capable of hypothesizing any type of relations and interactions among research variables in a single causal framework. This technique is helpful for researchers to better understand the concept of latent variables and their action within the model. This method has been employed in a wide range of studies, especially in food behaviour analysis and public health [36,37,38]. In this paper, three features of the SEM technique are presented:

#### 2.2.1. The Ability to Use Latent Variables

According to Bollen [39], “latent variables provide a degree of abstraction that permits us to describe relations among a class of events or variables that share something in common.” A specific characteristic of SEM is the use of “latent variables,” which are not applied in any other analysis method. Latent variables refer to constructs that are not directly observable. 

#### 2.2.2. The Ability to Estimate Direct and Indirect Effects

If the aim is to produce a research model based on regression modeling, the research framework will have the following structure:

(Family food security level + Parental socioeconomic status + Parental feeding behaviour + Child’s food intake) → Child’s weight(2)

**Figure 2:**
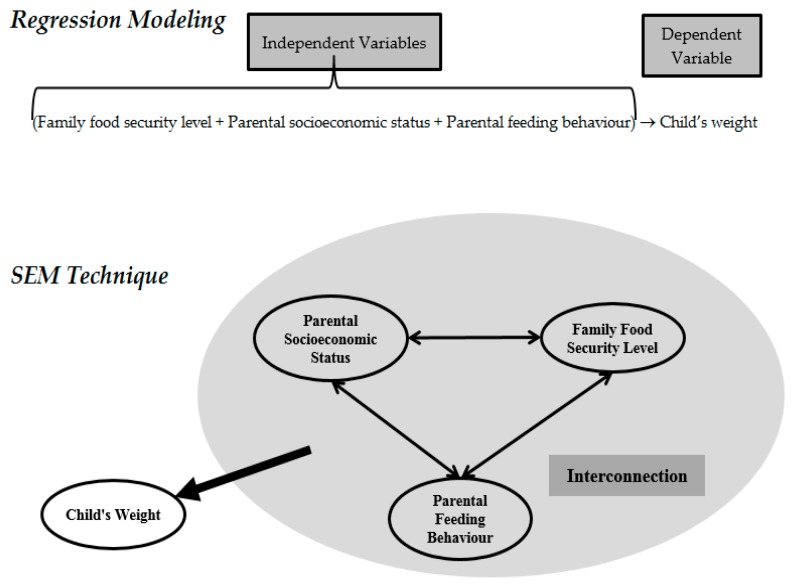
Structure of research variables in regression modeling with the SEM technique.

However, it is obvious that if there are any parental socioeconomic changes, the parents will try to change the internal family food security level in order to control the parental feeding behaviour and child’s food intake. The most significant advantage of SEM is the ability to simultaneously model and examine the indirect and direct interrelationships that exist among multiple dependent and independent variables [40] (see Figure 2).

#### 2.2.3. The Ability to Perform Simultaneous Estimation

Various statistical methods, such as multiple regression analysis, MANOVA, ANOVA, *t*-test and canonical correlation analysis have a common limitation. Most approaches can express a single link between the independent and dependent variables. In regression analysis, however, one or more independent variables are involved in a study, but there should be only one dependent variable. Canonical correlation analysis and MANOVA may involve more than one independent and dependent variables, but the analysis is restricted in that it can only display the relationship between independent and dependent variables. On the other hand, SEM can show the relationships among dependent variables. In SEM, more than one exogenous and endogenous variables are estimated simultaneously. The causal relationship between endogenous variables can also be estimated. For example, when a researcher wants to see the relationship of A → B → C → D, a total of four analyses should be conducted in multiple regression analysis. Nonetheless, simultaneous estimation is possible with SEM [41].

### 2.3. Sampling

In the present study, a cross-sectional research design is used. A cross-sectional research design applies any given research population sample at one point in time to obtain the required data. Moreover, the researcher cannot focus on development matters nor provide unsystematic interpretations. Hair, Black [42] suggested that the minimum sample size depends on model complexity and basic measurements of model characteristics. Therefore, we needed more than 300 respondents according to model characteristics with three latent constructs and perhaps after factor loading analysis, some of the constructs have less than three items (see Table 2).

In the present study, SEM was conducted for two subsets of data (normal weight group and overweight/obese group). The purpose of dividing into two groups was to know in the children group who has obesity issue and which factors have significant impact on child’s weight, in order to compare with the normal group. Based on the number of constructs in the research model (Figure 1) and Table 2 every group needed 100 samples.

There are 56 public primary schools with over 500 students located in the centre of Urumqi City (Xinjiang Province, China). The sampling procedure included three phases. The first phase was cluster sampling, whereby every primary school was considered one cluster. We contacted all schools by email and phone and requested cooperation for this research, and only eight confirmed willingness to participate. One of the schools was not ready on the date when data collection started, therefore seven schools were prepared for data collection. Ninety (90) questionnaires were delivered to every primary school (cluster). The second phase entailed stratified sampling. In this phase, every grade was defined as a strata. Each primary school includes six grades (six strata), and 15 questionnaires were considered for each grade. The third phase was random sampling from volunteer parents in every grade. Therefore, 7 × 6 × 15 = 630 questionnaires were considered the sampling number. We trained 25 bachelor students in public health and management to collect data and interview the parents based on the study survey. The volunteer parents were invited to the school by the school principals. We asked the parents to bring their children’s health cards. Each questionnaire took 15 to 20 min. Data collection lasted from 5 September 2016 to 20 October 2016. The survey was conducted with funding by the University of Malaya (project number RP027E-15HNE) and the University of Malaya Research Ethics Committee approved the research procedure (UM.TNC2/RC/H&E/UMREC 127). A parent was retained in the sample if they had a child between seven (grade one) and twelve (grade six) years of age.

Classical methods of standard SEM analysis focus on the sample covariance matrix. The AMOS software package has been developed on the basis of the covariance structure analysis approach with the sample covariance matrix [43]. Therefore, the AMOS’ 16 maximum likelihood program was used to examine the proposed hypothetical model.

## 3. Results

### 3.1. Descriptive Analysis

Table 3 and Table 4 represent the descriptive statistics of the sample and control variables’ characteristics in this study. Table 5 shows the BMI distribution among the observational variables in the study.

Based on Table 5, 12.86% children in the study are underweight, 63.81% are in the normal range and 23.34% (147) are in the overweight and obese ranges.

### 3.2. Study Reliability and Validity

Fornell and Larcker [44] defined questionnaire validity and reliability based on the following terms and conditions:

(a) Validity: Cronbach’s alpha of every latent variable must be equal to or higher than 0.7
(b) Reliability:
  - The average variance extracted (AVE) for every latent variable must be equal to or higher than 0.50
  - The factor loading of every indicator must be higher than 0.70 in the construct

Table 6 shows the outputs from the AVE and Cronbach’s alpha analysis. There are five groups of indicators. The first three groups are the research latent variables, family food security level (as a separate questionnaire) and some control variables with one dependent variable that were defined as a group of control variables. Table 6 illustrates that all research group variables have acceptable Cronbach’s alpha and AVE values.

Table 7 presents the factor loadings of the indicators on three research latent variables. As illustrated in this table, the factor loadings of eight indicators are less than 0.5; therefore, these indicators must be excluded from the measurement model. As a results, by excluding some indicators, the study reliability is confirmed.

### 3.3. Analysis of Model Fit

**Figure 3:**
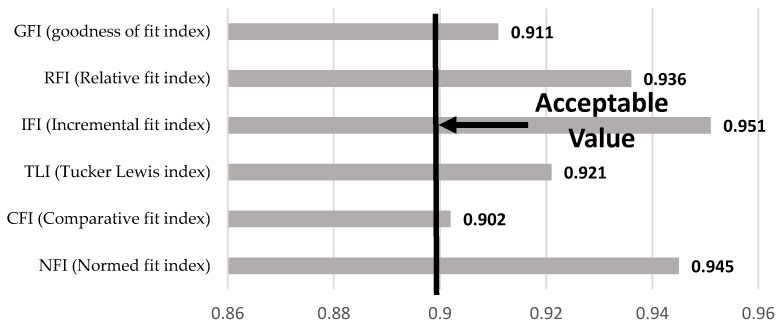
Model fit analysis.

Figure 3 shows the model fitting results based on the SEM approach. The GFI, RFI, IFI, TLI, CFI, and NFI values are within acceptable ranges. Therefore, the current model fits our data at the 5% significance level.

### 3.4. Normality Testing

Skewness and kurtosis are criteria that illustrate the normality or non-normality of every indicator. If the absolute kurtosis value is less than 7 and the value of skewness is between −2 and +2, the endogenous variables’ normality is acceptable. Table 8 presents the normality analysis of each research indicator after reliability analysis. 

The normality of all indicators individually is accepted by the skewness and kurtosis outputs. Moreover, based on the multivariate normality test output, the kurtosis value is 9.821. Since this value is less than 10 the multivariate normality is accepted [45].

### 3.5. Multicollinearity Analysis

**Figure 4:**
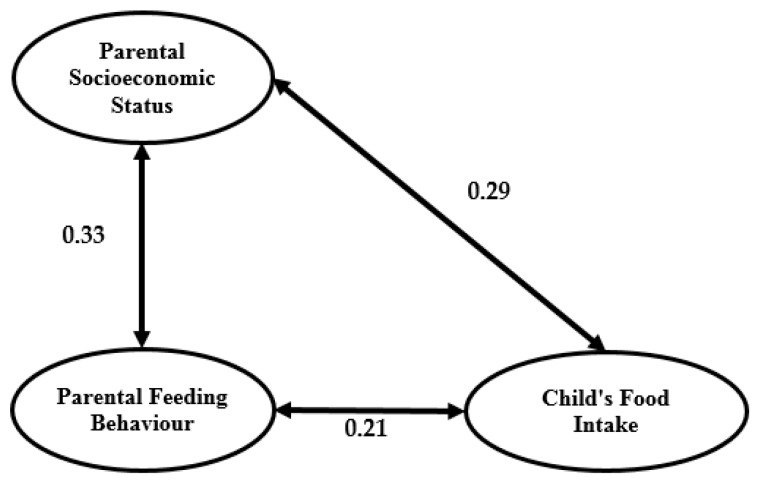
Full measurement model.

Multicollinearity is a serious problem in classical SEM modeling. Weak discriminant validity of the research model indicators usually causes multicollinearity in a study. In Figure 4, a double arrow represents the covariance among latent research variables. Kline and Klammer [46] determined that a high correlation between two latent constructs (greater than 0.85) signifies multicollinearity. Regarding the output in Figure 4, the correlation among the three latent constructs does not exceed 0.85, which confirms there is no multicollinearity in the research model.

### 3.6. Structural Model

**Figure 5:**
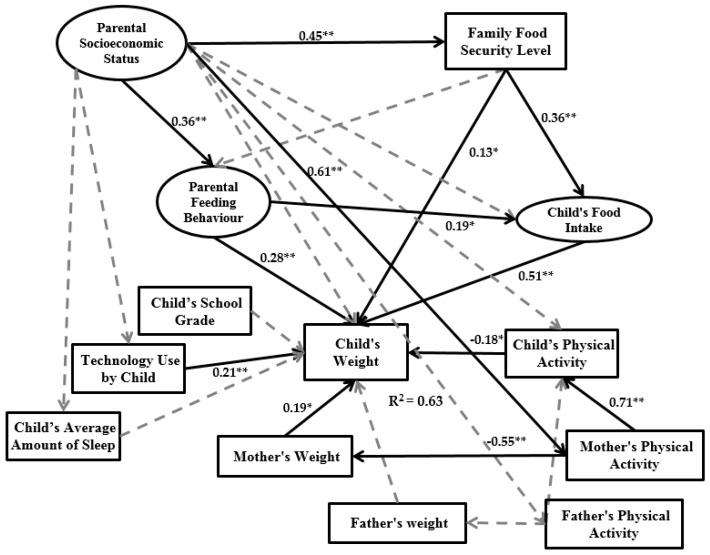
Normal model output (* Significant in the level of 5%; ** Significant in the level of 1%).

**Figure 6:**
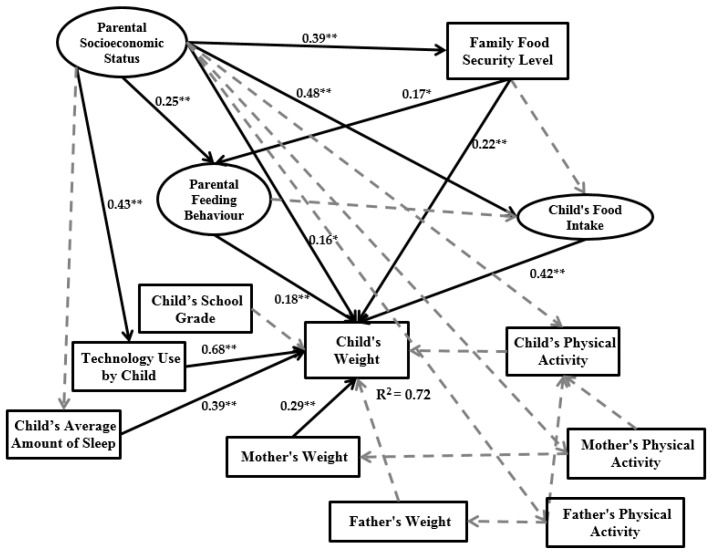
Obesity model output (* Significant in the level of 5%; ** Significant in the level of 1%).

A structural model was applied to identify the hypothesized connection among research constructs (exogenous or endogenous), which is linked to the assumed model’s concept. Based on Table 5, the BMI of 402 observations is in a normal range, and for 147 observations the child’s BMI ranges from overweight to obesity. Therefore, in this part of the study two structural models are presented: one based on the normal BMI range (hereby called a normal model) and one based on the obesity BMI range (hereby called an obesity model). Figure 5 and Figure 6 present structural models of the normal and obesity models.

The normal model results in Figure 5 demonstrate that parental socioeconomic status has a significant positive impact on family food security level, parental feeding behaviour and mother’s physical activity. However, the parental socioeconomic status variable does not have a significant impact on child’s food intake and weight. According to the obesity model in Figure 6, parental socioeconomic status has a significant impact on family food security level, child’s food intake, child’s weight and parental feeding behaviour. The family food security level in the normal model has significant impact on both child’s food intake and child’s weight, but no significant impact on parental feeding behaviour. In the obesity model, this variable only has significant impact on the child’s weight and parental feeding behaviour, and the impact of family food security level on the child’s food intake is not significant. In the normal model, parental feeding behaviour has significant impact on the child’s food intake and child’s weight. However, in the obesity model, parental feeding behaviour significantly affects only the child’s weight. Child’s food intake in both models has significant impact on the child’s weight. In the normal model, technology use by child, child’s physical activity and mother’s weight significantly impact the child’s weight. In the obesity model, the mother’s weight, technology use by child and child’s average amount of sleep have significant impact on the child’s weight.

## 4. Discussion

The main aim of this study was to examine a multi-factorial model of the relationship between household environment and childhood obesity by applying SEM. The child’s weight was the main dependent variable and parental socioeconomic status was the main independent variable. Between parental socioeconomic status and child’s weight, two latent variables and nine measurement variables were defined based on previous studies. Therefore, the introduced model was designed according to improvements on previous theories and frameworks of childhood obesity modeling based on family environment [21,33]. To evaluate the accuracy and validity of the final research model keeping in view suitable variables, sampling was done on Chinese families living in Urumqi (Xinjiang Province, China). The sample size was 630 families and data were collected between 5 September 2016 and 20 October 2016. The research framework is presented in Figure 1. We extracted two types of data from the dataset. The first group includes the normal BMI range and the second group is the obesity BMI range. The outputs of the two groups are presented in Figure 5 and Figure 6.

According to the findings, R^2^ in the obesity model is 0.72. This means that 72% of a child’s weight variation is dependent on parental socioeconomic status, family food security level, parental feeding behaviour, child’s food intake, technology use by child and child’s average amount of sleep, and the mother’s weight. However, this amount in the normal model is 63% with different significant variables.

In the normal model there is a strong interrelationship among parental socioeconomic status, parental feeding behaviour and child’s food intake as confirmed by Kröller and Warschburger [33] study. However, this interrelationship is broken by the non-significance impact of parental feeding behaviour on child’s food intake in the obesity model.

The impact of parental socioeconomic status on the child’s weight was confirmed by Crouch et al. [47], Keane et al. [48], and Walsh and Cullinan [49]. In their studies, all data included overweight, obesity, and normal BMI among the relationships. However, in the current study the data was categorized in two groups of normal and obesity concepts. Based on our data analysis, the impact of parental socioeconomic status on the child’s weight is significant in obesity modeling, which was not confirmed in the normal model. This indicates that in families who have obese children, their socioeconomic status has significant impact on their children’s weight. The mother’s education, father’s income and parents’ marriage length are confirmed to act as significant indicators of the parental socioeconomic status latent variable (Table 7), affecting the child’s weight in the obesity model.

In both normal and obesity models, the parental socioeconomic status has significant impact on family food security level. To elaborate further, higher-income parents (especially the father) or those with higher educational levels (especially the mother) seem more likely to be able to provide greater food security levels at home. Prior studies also support these results [50]. Moreover, our study showed that this relationship in the normal model (β = 0.45) is stronger than in the obesity model (β = 0.39). The output of this analysis reveals that families with a normal child BMI range have stronger plans with their socioeconomic status and their household food security level.

**Figure 7:**
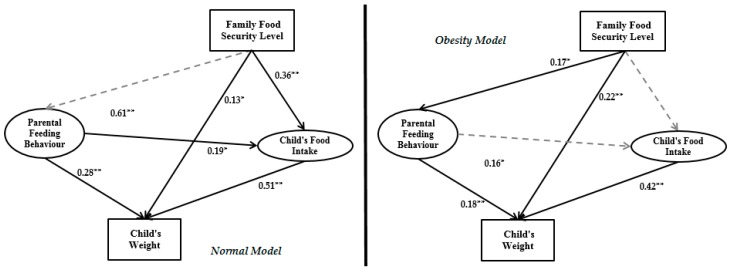
Impact of family food security level in the research framework for normal and obesity data (* Significant in the level of 5%; ** Significant in the level of 1%).

Family food security level in the obesity model has a significant impact on both parental feeding behaviour and the child’s weight. However, there is no significant impact on the child’s food intake. This relationship is different in the normal model, where the family food security level has a significant impact only on the child’s weight and child’s food intake but no significant impact on parental feeding behaviour (see Figure 7).

Most importantly, the path coefficient between family food security level and the child’s weight shows a significant relationship, which has been reported in earlier research works as well [51,52]. The results of the present research indicate that in spite of the direct relation between the child’s weight and socioeconomic situation, an indirect but meaningful relationship exists between them through family food security level, parental feeding behaviour and the child’s food intake.

**Figure 8:**
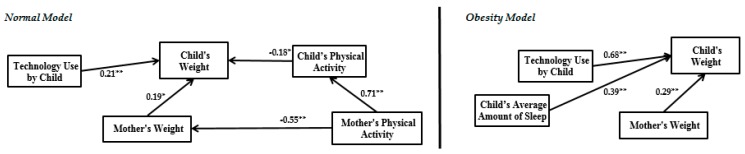
Structure of the impact of control variables on child’s weight in the obesity and normal models (* Significant in the level of 5%; ** Significant in the level of 1%).

The literature on childhood obesity emphasizes the key role of the average amount of sleep of children [53,54,55], physical activity [56,57], and technology use [58,59,60] in controlling childhood obesity. In our study, the impact of the control variables on child weight is shown in Figure 8.

The structure of the control variables’ impact on the dependent variable (child’s weight) in the obesity model is different than in the normal model. In both obesity and normal models, technology use by child has significant impact on the child’s weight. This impact in the obesity model (β = 0.68) is much higher than in the normal model (β = 0.21). The child’s average amount of sleep in the obesity model has a significant impact on the child’s weight. However, this impact is not significant in the normal model. Therefore, in the obesity model, Chinese primary school students who spend more time using devices including TV, video games, laptop/PC and mobile telephones and more time sleeping have a greater increase in weight than in the normal model. We found that technology use by children is the most effective risk factor compared to sleep time and physical activity in predicting child weight in obesity modeling.

It was illustrated that SEM analysis is a suitable technique for obesity modeling studies that has been used by other researchers [21,33]. However, the ordinary least squares (OLS) is a popular regression analysis method. For verification, we used four indices to compare OLS and SEM, which are representative of the strength and correctness of the prediction analysis. Root mean square error (RMSE), coefficient of determination (R^2^), mean absolute error (MSE) and mean absolute percentage error (MAPE) are the most familiar statistical indices for a comparison study among different prediction techniques. Table 9 presents the formula indices and output of OLS and SEM in both obesity and normal models.

## 5. Conclusions

This study on the complexity of parental socioeconomic status and family food security level with both parental feeding behaviour and child’s food intake that lead to child weight has a number of strengths:

(1) Improved previous studies relate parental socioeconomic status, parental feeding behaviour, child’s food intake and child’s weight by considering family food security level and some child environmental indicators like technology use by child and the child’s average amount of sleep.
(2) We illustrated that for childhood obesity analysis, especially for estimating child weight, obesity data should be extracted from the whole dataset and modeled separately. As seen in Figure 5 and Figure 6, there is a different output model structure between normal data and obesity data.

Although in the obesity model the child’s average amount of sleep and mother’s weight are significant, these variables are not significant in the normal model. Nevertheless, in the normal model, the interrelation among child’s physical activity, mother’s physical activity and mother’s weight is clear, which have significant impact on child weight. However, this interconnection is not significant in the obesity model. The main contribution of the current paper is thus illustrating a different final framework between the normal model and obesity model. The limitations of the current study lead to some suggestions for future studies as follows:

(1) This study was limited by the cross-sectional nature, which does not allow determining temporal relationships. We suggest doing this study with longitudinal data, which would provide researchers with more confidence in data analysis accuracy.
(2) In previous studies the child’s calorie intake [61] and genetics are deemed remarkable factors in causing obesity [62] and should be included in the model. We had limitations with collecting this type of data, so it is recommended to study them in future investigations on account of their significance.
(3) There are some indicators that can logically affect family environment and childhood obesity. These variables are economic, political and cultural determinants that cannot be measured based on the current research framework. However, they directly and indirectly have some impact on parental socioeconomic status, family food security level and parental feeding behaviour that lead to child weight. The data structure employed in this study is cross sectional and data were collected from Urumqi City, China. Therefore, there is one economic policy that controls the research model. This research model can thus be applied in other provinces of China and other countries, or comparison studies can be carried out among provinces or countries. In a comparison study, a moderating variable can be considered as an index of economic situation, which impacts all relationships among the research model variables.
(4) The research framework was designed based on children 7–12 years old and it is not suitable for children below primary school age. For future studies (obesity modeling for below 7 years old), some indicators like the child’s physical activity, child’s average amount of sleep, technology use by child, and child’s school grade should be excluded from the research variables in obesity modeling.

Finally, three recommendations for practice or policy-making to improve children’s weight and control childhood obesity are as follows:

(1) To measure and estimate a child’s weight in terms of obesity, it is better for practitioners to extract obesity data from the whole dataset.
(2) Family food security level should be addressed in future studies.
(3) The general knowledge of society should be increased regarding the high effect of child technology use and child’s average amount of sleep on children’s weight by presenting childhood obesity topics on TV shows, social media, and in primary school parental meetings.

## Figures and Tables

**Table 1:** BMI categories for children.

Category	BMI Range (kg/m^2^)
Underweight	<18.5
Normal Range	18.5–22.9
Overweight—At Risk	23.0–24.9
Overweight—Moderately Obese	25.0–29.9
Overweight—Severely Obese	≥30.0

**Table 2:** Minimum sample size required for SEM analysis.

Model Characteristics (Number of Latent Constructs and Items)	Minimum Sample Required
1. Five or less latent constructs. Each latent construct has more than three measurement items.	100 samples
2. Seven or less latent constructs. Each construct has more than three items.	150 samples
3. Seven or less latent constructs. Some constructs have less than three items (the identified model)	300 samples
4. More than seven latent constructs. Some constructs have less than three items (the identified model)	500 samples

**Table 3:** Descriptive statistics of family characteristics.

**Mother’s Age**	**Number**	**Percentage**	**Father’s Work Experience**	**Number**	**Percentage**
30 years old or younger	102	16.19%	Less than 5 years	61	9.68%
31 to 40 years old	254	40.32%	5–10 years	76	12.06%
41 to 50 years old	219	34.76%	10–15 years	196	31.11%
Over 50 years old	55	8.73%	15–20 years	228	36.19%
**Father’s Age**	**Number**	**Percentage**	More than 20 years	69	10.95%
30 years old or younger	74	11.75%	**Mother’s Education**	**Number**	**Percentage**
31 to 40 years old	186	29.52%	Less than High School	29	4.60%
41 to 50 years old	259	41.11%	High School	76	12.06%
Over 50 years old	111	17.62%	Diploma	212	33.65%
**Mother’s Income**	**Number**	**Percentage**	Bachelor	244	38.73%
Less than RMB2000	109	17.30%	Master or Ph.D.	69	10.95%
RMB2001–RMB3000	166	26.35%	**Father’s Education**	**Number**	**Percentage**
RMB3001–RMB4000	198	31.43%	Less than High School	39	6.19%
RMB4001–RMB5000	75	11.90%	High School	154	24.44%
More than RMB5000	26	4.13%	Diploma	269	42.70%
**Father’s Income**	**Number**	**Percentage**	Bachelor	102	16.19%
Less than RMB2000	88	13.97%	Master or Ph.D.	66	10.48%
RMB2001–RMB3000	206	32.70%	**Parents’ Marital Length**	**Number**	**Percentage**
RMB3001–RMB4000	195	30.95%	Less than 2 years	89	14.13%
RMB4001–RMB5000	66	10.48%	2–4 years	237	37.62%
More than RMB5000	75	11.90%	5–7 years	206	32.70%
**Mother’s Work Experience**	**Number**	**Percentage**	8–10 years	63	10.00%
Less than 5 years	66	10.48%	More than 10 years	35	5.56%
5–10 years	89	14.13%			
10–15 years	169	26.83%			
15–20 years	132	20.95%			
More than 20 years	133	21.11%			

**Table 4:** Descriptive statistics of the control variables in the study.

**Gender**	**Number**	**Percentage**	**Technology Use by Child**	**Number**	**Percentage**
Boy	286	45.39%	Less than one hour per day	86	13.65%
Girl	344	54.61%	1 to 2 h per day	186	29.52%
**Child’s School Grade**	**Number**	**Percentage**	3 to 4 h per day	208	33.02%
First (Seven years old)	105	16.67%	More than 4 h per day	150	23.81%
Second (Eight years old)	105	16.67%	**Child’s Average Sleep** **Duration**	**Number**	**Percentage**
Third (Nine years old)	105	16.67%			
Fourth (Ten years old)	105	16.67%	Less than 7 h per day	158	25.08%
Fifth (Eleven years old)	105	16.67%	7 to 8 h per day	296	46.98%
Sixth (Twelve years old)	105	16.67%	8 to 9 h per day	102	16.19%
**Mothers’ Physical Activity**	**Number**	**Percentage**	More than 9 h per day	74	11.75%
None	134	21.27%	**Fathers’ Physical Activity**	**Number**	**Percentage**
1 or 2 times per week	135	21.43%	None	298	47.30%
3 or 4 times per week	172	27.30%	1 or 2 times per week	186	29.52%
More than 4 times per week	189	30.00%	3 or 4 times per week	82	13.02%
**Child’s Physical Activity**	**Number**	**Percentage**	More than 4 times per week	64	10.16%
None	205	32.54%			
1 or 2 times per week	189	30.00%			
3 or 4 times per week	137	21.75%			
More than 4 times per week	99	15.71%			

**Table 5:** BMI distribution.

Category	Number (Percentage)
Underweight	81 (12.86%)
Normal Range	402 (63.81%)
Overweight—At Risk	82 (13.02%)
Overweight—Moderately Obese	41 (6.51%)
Overweight—Severely Obese	24 (3.81%)

**Table 6:** Results of average variance extracted (AVE) and Cronbach’s alpha.

Construct	AVE	Cronbach’s Alpha
Parental Socioeconomic Status	0.57	0.77
Parental Feeding Behaviour	0.61	0.71
Child’s Food Intake	0.61	0.81
Family Food Security Level	Not Applicable	0.78
Group of control variables	Not Applicable	0.76

**Table 7:** Factor loading analysis of research latent variables.

Parameter Description	Factor Loading
*Parental Socioeconomic Status*	
Mother’s education	0.86
Father’s education	0.44
Mother’s income	0.48
Father’s income	0.73
Mother’s work experience	0.21
Father’s work experience	0.33
Parents’ marriage length	0.92
*Parental Feeding Behaviour*	
Rewarding	0.48
Restricting	0.72
Pressuring	0.81
Modeling	0.47
Controlling	0.77
Monitoring	0.36
*Child’s Food Intake*	
Sweets	0.89
Chips	0.92
Soft Drinks	0.96
Fruits	0.57
Vegetables	0.56
Fast Food	0.66
Whole Grains	0.41

**Table 8:** Normality test.

Indicators	Skew	Kurtosis
Mother’s education	1.018	0.581
Father’s income	0.658	−0.324
Parents’ marriage length	−0.578	−0.207
Household food security level	1.971	6.325
Child technology use	1.598	2.059
Child’s average amount of sleep	0.982	1.297
Child’s weight	0.624	2.125
Child’s physical activity	−0.259	−0.657
Mother’s physical activity	−0.597	−0.957
Father’s physical activity	−1.287	−4.268
Mother’s weight	0.951	2.687
Father’s weight	1.058	3.059
Restricting	0.663	−0.411
Pressuring	0.288	−1.014
Controlling	1.698	0.586
Sweets	0.886	−1.185
Chips	0.444	0.742
Soft drinks	1.051	−1.004
Fast food	0.222	1.196
Vegetables	0.875	0.201

**Table 9:** Comparative outputs of SEM and OLS in obesity and normal models.

Formula	SEM (Obese)	SEM (Normal)	OLS (Obese)	OLS (Normal)
$MAPE=\frac{1}{n}\sum_{i=1}^{n}\left|\frac{y_{i}^{\prime }-y_{i}}{y_{i}}\right|$	0.987	1.485	3.688	2.598
$RMSE=\sqrt[{2}]{\frac{\sum_{i=1}^{n}{\left(y_{i}^{\prime }-y_{i}\right)}^{2}}{n}}$	1.157	2.014	3.894	3.996
$MSE=\frac{\sum_{i=1}^{n}\left|y_{i}^{\prime }-y_{i}\right|}{n}$	1.269	2.229	4.597	7.071
$R^{2}=\frac{{\left[\sum_{i=1}^{n}\left(y_{i}^{\prime }-{\bar{y}}_{i}^{,}\right)\cdot \left(y_{i}-{\bar{y}}_{i}\right)\right]}^{2}}{\sum_{i=1}^{n}\left(y_{i}^{\prime }-{\bar{y}}_{i}^{\prime }\right)\cdot \sum_{i=1}^{n}\left(y_{i}-{\bar{y}}_{i}\right)}$	0.72	0.63	0.61	0.55

Where $y_{i}$ is the *i*th actual value of the dependent variable and $y_{i}^{\prime }$ is the *i*th predicted value. The R^2^ value for SEM in both models was greater than OLS, and the MAPE, RMSE and MSE values of the SEM outputs were lower than OLS. Therefore, the performance indices with SEM are better in predicting child weight than the OLS model.

## Appendix A

**Table A1:** 18-Question Core Food Security Module.

1	“We worried whether our food would run out before we got money to buy more.” Was that often, sometimes, or never true for you in the last 12 months?	
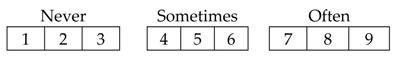
2	“The food that we bought just didn’t last and we didn’t have money to get more.” Was that often, sometimes, or never true for you in the last 12 months?	
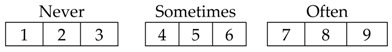
3	“We couldn’t afford to eat balanced meals.” Was that often, sometimes, or never true for you in the last 12 months?	
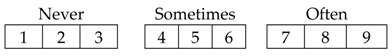
4	In the last 12 months, did you or other adults in the household ever cut the size of your meals or skip meals because there wasn’t enough money for food? (Yes/No)	
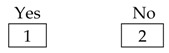
5	(If yes to Question 4) How often did this happen almost every month, some months but not every month, or in only 1 or 2 months?	
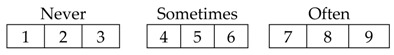
6	In the last 12 months, did you ever eat less than you felt you should because there wasn’t enough money for food? (Yes/No)	
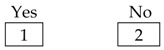
7	In the last 12 months, were you ever hungry, but didn’t eat, because there wasn’t enough money for food? (Yes/No)	
8	In the last 12 months, did you lose weight because there wasn’t enough money for food? (Yes/No)	
9	In the last 12 months did you or other adults in your household ever not eat for a whole day because there wasn’t enough money for food? (Yes/No)	
10	(If yes to Question 9) How often did this happen almost every month, some months but not every month, or in only 1 or 2 months?	
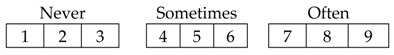
11	“We relied on only a few kinds of low-cost food to feed our children because we were running out of money to buy food.” Was that often, sometimes, or never true for you in the last 12 months?	
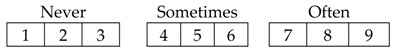
12	“We couldn’t feed our children a balanced meal, because we couldn’t afford that.” Was that often, sometimes, or never true for you in the last 12 months?	
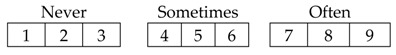
13	“The children were not eating enough because we just couldn’t afford enough food.” Was that often, sometimes, or never true for you in the last 12 months?	
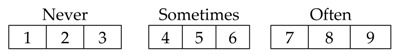
14	In the last 12 months, did you ever cut the size of any of the children’s meals because there wasn’t enough money for food? (Yes/No)	
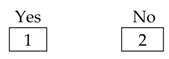
15	In the last 12 months, were the children ever hungry but you just couldn’t afford more food? (Yes/No)	
16	In the last 12 months, did any of the children ever skip a meal because there wasn’t enough money for food? (Yes/No)	
17	(If yes to Question 16) How often did this happen almost every month, some months but not every month, or in only 1 or 2 months?	
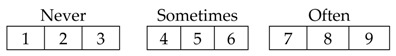
18	In the last 12 months, did any of the children ever not eat for a whole day because there wasn’t enough money for food? (Yes/No)	

## References

[1] Sun H., Ma Y., Han D., Pan C.W., Xu Y. (2014). Prevalence and trends in obesity among china’s children and adolescents, 1985–2010. PLoS ONE.

[2] Martinson M.L., Chang Y.L., Han W.J., Wen J. (2015). Social determinants of childhood obesity in Shanghai, China: A cross-sectional child cohort survey. Lancet.

[3] Zhang Y.X., Wang S.R. (2011). Changes in nutritional status of children and adolescents in Shandong, China from 1995 to 2005. Ann. Hum. Biol..

[4] Zou Y., Zhang R.H., Xia S.C., Huang L.C., Fang Y.Q., Meng J., Chen J., Zhang H.-X., Zhou B., Ding G.Q. (2016). The Rural-Urban Difference in BMI and Anemia among Children and Adolescents. Int. J. Environ. Res. Public Health.

[5] Commission on Ending Childhood Obesity (2016). Report of the Commission on Ending Childhood Obesity.

[6] Vinturache A.E., McDonald S., Slater D., Tough S. (2015). Perinatal outcomes of maternal overweight and obesity in term infants: A population-based cohort study in Canada. Sci. Rep..

[7] Baek S.H., Chung H.J., Lee H.K., D’Souza R., Jeon Y., Kim H.J., Kweon S.J., Hong S.T. (2014). Treatment of obesity with the resveratrol-enriched rice DJ-526. Sci. Rep..

[8] Kaur J., Lamb M.M., Ogden C.L. (2015). The association between food insecurity and obesity in children—The National Health and Nutrition Examination Survey. J. Acad. Nutr. Diet..

[9] Mosli R.H., Miller A.L., Peterson K.E., Kaciroti N., Rosenblum K., Baylin A., Lumeng J.C. (2016). Birth order and sibship composition as predictors of overweight or obesity among low-income 4- to 8-year-old children. Pediatr. Obes..

[10] Doub A.E., Small M., Birch L.L. (2016). A call for research exploring social media influences on mothers’ child feeding practices and childhood obesity risk. Appetite.

[11] Raynor H.A., Van Walleghen E.L., Osterholt K.M., Hart C.N., Jelalian E., Wing R.R., Goldfield G.S. (2011). The relationship between child and parent food hedonics and parent and child food group intake in children with overweight/obesity. J. Am. Diet. Assoc..

[12] Mandal B., Powell L.M. (2014). Child care choices, food intake, and children’s obesity status in the United States. Econ. Hum. Biol..

[13] Andrews K.R., Silk K.S., Eneli I.U. (2010). Parents as health promoters: A theory of planned behavior perspective on the prevention of childhood obesity. J. Health Commun..

[14] Ball K., Mishra G., Crawford D. (2002). Which aspects of socioeconomic status are related to obesity among men and women?. Int. J. Obes..

[15] Nau C., Schwartz B.S., Bandeen-Roche K., Liu A., Pollak J., Hirsch A., Lisa B.D., Glass T.A. (2015). Community socioeconomic deprivation and obesity trajectories in children using electronic health records. Obesity.

[16] Sobal J., Stunkard A.J. (1989). Socioeconomic status and obesity: A review of the literature. Psychol. Bull..

[17] Nichols M.S., de Silva-Sanigorski A.M., Cleary J.E., Goldfeld S.R., Colahan A., Swinburn B.A. (2011). Decreasing trends in overweight and obesity among an Australian population of preschool children. Int. J. Obes..

[18] Patrick H., Nicklas T.A., Hughes S.O., Morales M. (2005). The benefits of authoritative feeding style: Caregiver feeding styles and children’s food consumption patterns. Appetite.

[19] Clark H.R., Goyder E., Bissell P., Blank L., Peters J. (2007). How do parents’ child-feeding behaviours influence child weight? Implications for childhood obesity policy. J. Public Health.

[20] Faith M.S., Scanlon K.S., Birch L.L., Francis L.A., Sherry B. (2004). Parent-child feeding strategies and their relationships to child eating and weight status. Obesity.

[21] Hendrie G.A., Coveney J., Cox D.N. (2012). Defining the complexity of childhood obesity and related behaviours within the family environment using structural equation modelling. Public Health Nutr..

[22] Boles R.E., Halbower A.C., Daniels S., Gunnarsdottir T., Whitesell N., Johnson S.L. (2016). Family Chaos and Child Functioning in Relation to Sleep Problems Among Children at Risk for Obesity. Behav. Sleep Med..

[23] Shochat T., Cohen-Zion M., Tzischinsky O. (2014). Functional consequences of inadequate sleep in adolescents: A systematic review. Sleep Med. Rev..

[24] Chen X., Beydoun M.A., Wang Y. (2008). Is sleep duration associated with childhood obesity? A systematic review and meta-analysis. Obesity.

[25] Wethington H., Pan L., Sherry B. (2013). The Association of Screen Time, Television in the Bedroom, and Obesity Among School-Aged Youth: 2007 National Survey of Children’s Health. J. Sch. Health.

[26] Jiang M.H., Yang Y., Guo X.F., Sun Y.X. (2013). Association between child and adolescent obesity and parental weight status: A cross-sectional study from rural north China. J. Int. Med. Res..

[27] Soos K.J. Assessing the Impact of a Parental Modeling Physical Acitivity Intervention on Child Physical Activity and Obesity Levels. http://thescholarship.ecu.edu/bitstream/handle/10342/5612/SOOS-HONORSTHESIS-2016.pdf?sequence=1&isAllowed=y.

[28] Finucane M.M., Stevens G.A., Cowan M.J., Danaei G., Lin J.K., Paciorek C.J., Singh G.M., Gutierrez H.R., Lu Y., Farzadfar F. (2011). National, regional, and global trends in body-mass index since 1980: Systematic analysis of health examination surveys and epidemiological studies with 960 country-years and 9.1 million participants. Lancet.

[29] Maddah M., Nikooyeh B. (2010). Factors associated with overweight in children in Rasht, Iran: Gender, maternal education, skipping breakfast and parental obesity. Public Health Nutr..

[30] Ogden C.L., Carroll M.D., Kit B.K., Flegal K.M. (2012). Prevalence of obesity and trends in body mass index among US children and adolescents, 1999–2010. JAMA.

[31] Bickel G., Nord M., Price C., Hamilton W., Cook J. (2000). Guide to Measuring Household Food Security in the United States.

[32] Birch L.L., Fisher J.O., Grimm-Thomas K., Markey C.N., Sawyer R., Johnson S.L. (2001). Confirmatory factor analysis of the Child Feeding Questionnaire: A measure of parental attitudes, beliefs and practices about child feeding and obesity proneness. Appetite.

[33] Kröller K., Warschburger P. (2009). Maternal feeding strategies and child’s food intake: Considering weight and demographic influences using structural equation modeling. Int. J. Behav. Nutr. Phys. Act..

[34] Song P., Li X., Gasevic D., Flores A.B., Yu Z. (2016). BMI, Waist Circumference Reference Values for Chinese School-Aged Children and Adolescents. Int. J. Environ. Res. Public Health.

[35] CDCP (Centers for Disease Control and Prevention) About BMI for Children and Teens. https://www.cdc.gov/healthyweight/assessing/bmi/childrens_bmi/about_childrens_bmi.html.

[36] Sarmugam R., Worsley A. (2015). Dietary behaviours, impulsivity and food involvement: Identification of three consumer segments. Nutrients.

[37] Tanja T.T., Outi N., Sakari S., Jarmo L., Kaisa P., Leila K. (2015). Preliminary finnish measures of eating competence suggest association with health-promoting eating patterns and related psychobehavioral factors in 10–17 year old adolescents. Nutrients.

[38] Wan Mohamed Radzi C.W.J.B., Hui H., Salarzadeh Jenatabadi H. (2016). Comparing Bayesian and Maximum Likelihood Predictors in Structural Equation Modeling of Children’s Lifestyle Index. Symmetry.

[39] Bollen K.A. (2002). Latent variables in psychology and the social sciences. Annu. Rev. Psychol.

[40] Gefen D., Straub D., Boudreau M.C. (2000). Structural equation modeling and regression: Guidelines for research practice. Commun. Assoc. Inf. Syst..

[41] Jeon J. (2015). The Strengths and Limitations of the Statistical Modeling of Complex Social Phenomenon: Focusing on SEM, Path Analysis, or Multiple Regression Models. Int. J. Soc. Behav. Educ. Econ. Bus. Ind. Eng..

[42] Hair J.F., Black W.C., Babin B.J., Anderson R.E., Tatham R.L. (2010). Multivariate Data Analysis.

[43] Lee S.Y. (2007). Structural Equation Modeling: A Bayesian Approach.

[44] Fornell C., Larcker D.F. (1981). Evaluating structural equation models with unobservable variables and measurement error. J. Mark. Res..

[45] Wan Mohamed Radzi C.W.J.B., Salarzadeh Jenatabadi H., Hasbullah M.B. (2015). Firm Sustainability Performance Index Modeling. Sustainability.

[46] Kline T.J., Klammer J.D. (2001). Path model analyzed with ordinary least squares multiple regression versus LISREL. J. Psychol. Interdiscip. Appl..

[47] Crouch P., O’dea J.A., Battisti R. (2007). Child feeding practices and perceptions of childhood overweight and childhood obesity risk among mothers of preschool children. Nutr. Diet..

[48] Keane E., Layte R., Harrington J., Kearney P.M., Perry I.J. (2012). Measured parental weight status and familial socio-economic status correlates with childhood overweight and obesity at age 9. PLoS ONE.

[49] Walsh B., Cullinan J. (2015). Decomposing socioeconomic inequalities in childhood obesity: Evidence from Ireland. Econ. Hum. Biol..

[50] Chi D.L., Dinh M.A., da Fonseca M.A., Scott J.M., Carle A.C. (2015). Dietary Research to Reduce Children’s Oral Health Disparities: An Exploratory Cross-Sectional Analysis of Socioeconomic Status, Food Insecurity, and Fast-Food Consumption. J. Acad. Nutr. Diet..

[51] Barroso C.S., Roncancio A., Moramarco M.W., Hinojosa M.B., Davila Y.R., Mendias E., Reifsnider E. (2016). Food security, maternal feeding practices and child weight-for-length. Appl. Nurs. Res..

[52] Solorio C.M.G. (2013). Maternal Food Insecurity, Child Feeding Practices, Weight Perceptions and BMI in a Rural, Mexican-Origin Population. Ph.D. Thesis.

[53] Miller A.L., Lumeng J.C., LeBourgeois M.K. (2015). Sleep patterns and obesity in childhood. Curr. Opin. Endocrinol. Diabetes Obes..

[54] Zhang J., Jin X., Yan C., Jiang F., Shen X., Li S. (2015). Short sleep duration as a risk factor for childhood overweight/obesity: A large multicentric epidemiologic study in China. Sleep Health.

[55] Boin A.C., Nozoe K.T., Polesel D.N., Andersen M.L., Tufik S. (2014). The possible influence of sleep in childhood obesity. Eur. J. Clin. Nutr..

[56] Nemet D. (2016). Childhood Obesity, Physical Activity, and Exercise. Pediatr. Exerc. Sci..

[57] Braden A., Strong D., Crow S., Boutelle K. (2015). Parent changes in diet, physical activity, and behavior in family-based treatment for childhood obesity. Clin. Pediatr..

[58] Walton K., Simpson J.R., Darlington G., Haines J. (2014). Parenting stress: A cross-sectional analysis of associations with childhood obesity, physical activity, and TV viewing. BMC Pediatr..

[59] Arora T., Hosseini-Araghi M., Bishop J., Yao G.L., Thomas G.N., Taheri S. (2013). The complexity of obesity in UK adolescents: Relationships with quantity and type of technology, sleep duration and quality, academic performance and aspiration. Pediatr. Obes..

[60] Silverstone S., Teatum J. (2011). Technology: The problem or the solution to childhood obesity. Am. J. Bus. Educ..

[61] Sinaga H.T., Doloksaribu B., Tobing H.M., Nurhayati I. (2016). Using scores in interpreting growth status effectively improved infant feeding practices and calorie intake of child aged 0–12 months. Int. J. Med. Sci. Public Health.

[62] Wardle J., Carnell S., Haworth C.M., Plomin R. (2008). Evidence for a strong genetic influence on childhood adiposity despite the force of the obesogenic environment. Am. J. Clin. Nutr..

